# Effect of Post-Printing Conditions on the Mechanical and Optical Properties of 3D-Printed Dental Resin

**DOI:** 10.3390/polym16121713

**Published:** 2024-06-15

**Authors:** Lippo Lassila, Enas Mangoush, Jingwei He, Pekka K. Vallittu, Sufyan Garoushi

**Affiliations:** 1 Department of Biomaterials Science and Turku Clinical Biomaterial Center—TCBC, Institute of Dentistry, University of Turku, 20520 Turku, Finland; liplas@utu.fi (L.L.); msjwhe@scut.edu.cn (J.H.); pekval@utu.fi (P.K.V.); sufgar@utu.fi (S.G.); 2 College of Materials Science and Engineering, South China University of Technology, Guangzhou 510640, China; 3 Wellbeing Services County of South-West Finland, 20014 Turku, Finland

**Keywords:** 3D-printed resins, post-printing conditions, flexural strength, surface wear, optical properties

## Abstract

This study aimed to evaluate the flexural strength (FS), surface wear, and optical properties of 3D-printed dental resins subjected to different post-printing conditions. A total of 240 specimens (2 × 2 × 25 mm³) were 3D-printed using resin materials for permanent (VaresoSmile Crown Plus) VSC and temporary (VaresoSmile Temp) VST restorations. Specimens underwent five post-printing conditions: no post-printing cure; post-cured in a Form Cure curing unit; Visio Beta Vacuum; Ivoclar Targis; or heat-cured (150 °C) for 30 min. Each group of specimens (*n* = 24) was tested either directly after post-curing, after 24 h of dry storage, or following hydrothermal accelerated aging in boiling water for 16 h. The three-point bending test was used to evaluate the FS. The two-body wear test was performed on 50 disc-shaped specimens (*n* = 5/group). Surface gloss and translucency were measured for permanent VSC specimens (*n* = 5/group). SEM/EDS and statistical analyses were performed. The Form Cure device yielded the highest FS and lowest wear depth (*p* < 0.05). Hydrothermal aging significantly reduced FS. There were no statistical differences in FS and wear values between materials subjected to same post-printing conditions. VSC groups exhibited similar optical properties across different post-printing treatments. Post-printing treatment conditions had a significant impact on the FS and wear of the 3D-printed resin, while optical properties remained unaffected.

## 1. Introduction

The widespread adoption of three-dimensional (3D) printing technology has become apparent across diverse medical fields. In light of the recent introduction of this technology to dentistry, there still are challenges to overcome, particularly in relation to material properties and their long-term clinical efficacy [1,2]. Three-dimensional printing is linked to diminished strength and fitting accuracy when compared with subtractive or milling manufacturing products [3]. However, this technology offers numerous advantages, it minimizes heat, noise emissions, and waste in terms of construction material and the need for machine bur replacements. Additionally, it makes it possible to produce small structures with high complexity. The versatility of 3D printing extends to various applications, including the fabrication of surgical and occlusal devices, implants, dentures, maxillofacial prostheses, and restorations [1,4]. This rapid emergence of 3D printing techniques and products, both commercially and in the scientific literature, highlights the necessity for a valid classification approach. One such approach involves categorizing contemporary 3D printing methods based on their fabrication processes [4]. Consequently, 3D printing methods fall into different categories, including digital light processing (DLP), stereolithography (SLA), selective laser sintering (SLS), inkjet printing, and fused deposition modeling (FDM) [5,6]. Each of these 3D printing methods has its advantages and limitations, and the choice of method depends on factors such as the desired application and resolution requirements. DLP is the most widely applied 3D printing technique in dentistry. The method operates through the utilization of a UV projector; this projector solidifies a photopolymer resin, layer by layer, by curing specific regions in accordance with the 3D printer model, while leaving the surrounding regions uncured. After completing one layer, the part is raised by one layer height (e.g., 50 or 100 µm), and the process repeats. Unlike other techniques, a whole layer of the pattern is cured instantly and simultaneously through a single projection of the entire layer, making it notably fast [7,8].

Various 3D printing resins intended for the fabrication of temporary and permanent restorations have recently emerged. Evidence suggests that 3D-printed resin crowns exhibit favorable mechanical properties, rendering them suitable for use as both temporary and permanent restorations [9]. However, due to the recent introduction of these materials into clinical use, there remains a lack of information regarding their structures and various properties. Moreover, the effect of different post-printing conditions on the performance of these materials is not yet well understood. 

VarseoSmile Temp (BEGO, Bremen, Germany) is a light-cured tooth-colored resin based on methacrylic acid esters for the 3D printing of temporary crowns and bridges, inlays, onlays, and veneers. According to the manufacturer, this temporary material is suitable for a broad range of applications. For instance, it is suitable for constructing up to a seven-unit bridge with a single pontic, and it can be utilized as an aesthetic temporary restoration for periods of up to 12 months [10]. A novel material, VarseoSmile Crown Plus (VSC), has been recently produced by the same manufacturer. It is specifically formulated to produce permanent tooth-colored restorations, such as full crowns, inlays, onlays, and veneers intended for single-tooth restorations. To the best of the authors’ knowledge, it is one of the first 3D-printed materials approved for permanent dental restorations, marking a significant advancement in the dental biomaterials field [10]. The composition of these materials places them within the category of a ceramic infiltrated hybrid composite, since they feature a matrix consisting of methacrylic ester with ceramic fillers, aligning them with the classification of a resin matrix ceramic [10]. According to the manufacturer, VSC and VST offer a range of advantages. Their composition ensures precision, easy handling, and diverse applications with low material costs, clear radiographic visibility, and biocompatibility with the surrounding environment [11]. 

Recent scientific papers predominantly focus on exploring the properties of 3D-printed materials mainly in the context of provisional restorations. Given the novelty of the tested permanent material, it is essential to bridge the existing literature gap. Crucial properties such as flexural strength (FS), modulus (FM), surface wear, and hardness are not comprehensively documented at present. This emphasizes the need for further investigations into the effect of certain printing designs and parameters, such as post-printing conditions, on the determination of the material properties. For instance, previous research has indicated that employing different printing directions and printing-layer thickness leads to notable variations in the properties of 3D printing restorations [12,13]. Therefore, this study aims to enhance the understanding of the applicability and limitations of 3D printing materials tested by evaluating the impact of post-printing treatment conditions, including different light-curing systems, heat curing, and hydrothermal accelerated aging, on the FS, surface wear, and optical properties such as surface gloss, translucency, and light transmission of the tested materials. Moreover, the chemical composition provided by the manufacturers is the same for both materials. Thus, a comparative evaluation of the tested properties and chemical composition of the two materials is conducted to identify any variations that might justify their classification as either temporary or permanent. 

The null hypothesis tested proposed that post-printing treatment conditions would not significantly alter the FS, surface wear, and optical properties of the materials under examination. 

## 2. Materials and Methods

### 2.1. Specimen Preparation 

A digital light processing (DLP) printer (Asiga MAX™, SCHEU-DENTAL GmbH, Iserlohn, Germany) was used to generate specimens from the evaluated commercial dental resins (Table 1), which were designed for printing permanent VSC and temporary VST restorations. The specimens were designed using Asiga Composer 2.0 software. This software allowed importing and adjusting of the 3D model as well as setting printing parameters such as layer thickness, print speed, and curing times. The print file was then transferred to the 3D printer to start the printing process. Each batch consisted of 25 specimens, ensuring efficient use of the printer’s build platform while maintaining controlled production standards. The printing was performed at 90° to the building platform, with a layer thickness of 100 μm. All specimens were cleaned in an ultrasonic bath (Quantrex^®^ 90, L&R Ultrasonics, Kearny, NJ, USA) filled with 97% isopropanol for 10 min and then dried with compressed air. Then, specimens were subjected to five distinct post-printing conditions: no post-printing cure; post-cured in a Form Cure curing unit (Formlabs, Berlin, Germany) for 30 min at 60 °C; post-cured in a Visio Beta Vario curing unit (3M ESPE, St. Paul, MN, USA) for 30 min at 40 °C; post-cured in an Ivoclar Targis Power curing unit (Ivoclar Vivadent AG, Schaan, Liechtenstein, Liechtenstein) for 30 min at 40 °C; heat-cured in an oven (Heraeus, Hanau, Germany) for 30 min at 150 °C. The curing temperatures used are recommended by the manufacturers for the used materials.

### 2.2. Flexural Strength Measurement 

To evaluate the FS (MPa) in accordance with ISO 4049 [14], a 3-point bending test was conducted on 240 bar-shaped specimens (2 × 2 × 25 mm^3^); specimens were measured using a digital caliper to ensure the uniformity of the specimens’ dimensions. This testing was carried out using a universal testing machine (Model LRX; Lloyds Instruments Ltd., Hampshire, UK) under ambient conditions at room temperature. The testing setup included a load cell with a capacity of 2500 N, a crosshead speed of 1 mm/min, an indenter with a diameter of 2 mm, and a 20 mm distance between the supports of the tested specimens. The endpoint of each test was the breaking point of the specimen.

A total of 24 specimens per group were equally divided and subjected to testing under different conditions. This included testing directly after post-curing (control), after 24 h of dry storage in an oven at 37 °C, and following hydrothermal accelerated aging in boiling water for 16 h. FS (ơf) was calculated using Formula (1):

ơf = 3F_m_I/(2bh^2^) (1)

where F_m_ is the applied load (N) at the highest point of the load-deflection curve, I is the span length (20 mm), b is the width of the test specimens, and h is the thickness of the test specimens.

### 2.3. Wear Resistance Measurement

**Figure 1:**
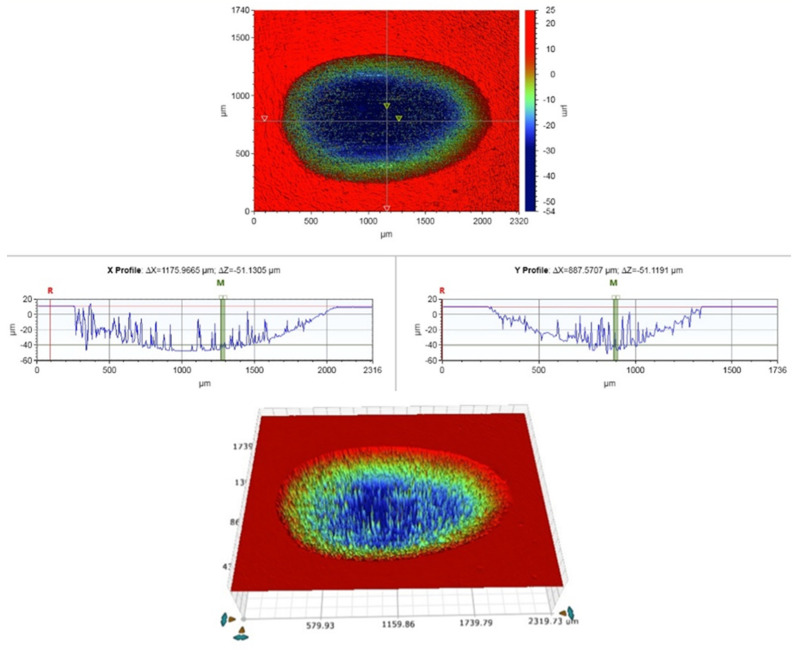
Surface profile of the wear pattern using a 3D optical microscope.

A two-body wear test was performed on disc-shaped (10 mm in diameter and 2 mm in thickness) specimens (*n* = 5/group). Each specimen was fixed to an acrylic resin block and sequentially polished using silicon carbide sheets with grain sizes up to 4000 grit FEPA. The specimens were stored in water at 37 °C for 24 h before testing. A chewing simulator (CS-4.2, SD Mechatronik, Feldkirchen-Westerham, Germany) with two chambers was used to conduct the wear test in the presence of water. The specimens were fixed to the lower plastic holder of the simulator, while the manufacturer’s standard loading tips (Steatite ball, 6 mm) were secured to the upper one with a fastening screw. A chewing simulation was performed at 1.5 Hz with a vertical weight of 2 kg, which is equivalent to 20 N of chewing force. Each specimen was subjected to 15,000 loading cycles. The wear patterns were then scanned with a 3D optical microscope (Bruker Nano GmbH, Berlin, Germany), and the material loss estimates were calculated using Vision64 Map software (version 1, Bruker Nano GmbH, Berlin, Germany) The total vertical wear depth values were acquired in micrometers (μm) from several sites by averaging the deepest points of all profile scans (Figure 1).

### 2.4. Surface Gloss Measurement

The surface gloss of polished disc-shaped resin specimens (*n* = 5/group) made of VSC was measured at a 60° incidence angle using a calibrated infrared Zehntner-Glossmeter with a measuring window size of 4.7 × 2 mm (GmbH Testing Instruments, Solingen, Germany). An average of three measurements per specimen was recorded. Polishing was conducted with various silicon carbide paper grit sizes (G1: 320) → (G2: 800) → (G3: 1200) → (G4: 2000) → (G5: 4000) at 300 rpm, under water cooling, using an automatic polishing machine (Struers Rotopol-11, Copenhagen, Denmark) for 1 min. The results were expressed in Gloss Units (GUs).

### 2.5. Translucency Parameter Measurement (TP)

The translucency parameter was calculated using the same polished disc-shaped (2 mm thickness) VSC specimens (*n* = 5/group) as used for gloss measurement. The CIELAB color scale was used to determine the specimens’ color relative to the standard illuminant D65. Color assessment was conducted on a reflection spectrophotometer (CM-700d, Konica-Minolta, Tokyo, Japan) over a black tile (CIE L* = 0, a* = 0.01, and b* = 0.03) and a white one (CIE L* = 99.25, a* = −0.09, and b* = 0.05). Aperture diameter was 3 mm, and the viewing and illuminating configuration was CIE diffuse/10° geometry with specular component included (SCI) geometry. To obtain the translucency of the tested composite specimens, the color difference between the specimen on the white background and the specimen on the black one was measured using Equation (2):

TP = [(LW* − LB*)2 + (aW* − aB*)2 + (bW* − bB*)2]^1/2^ (2)

where TP is the translucency parameter, the variable ‘W’ refers to color coordinates on the white background, and the variable ‘B’ refers to color coordinates on the black background.

### 2.6. Light Transmission Analysis

For composites made of VSC, three different material thicknesses (0.5, 1, and 2 mm) were taken (*n* = 3/thickness). A total of 45 disc specimens were prepared (*n* = 15/group). Every specimen was flat polished using an automatic grinding machine (Rotopol-1; Struers) and silicon carbide papers from #1200- to #4000-grit at 300 rpm under water cooling. A digital caliper (Mitutoyo Corp, Kanagawa, Japan) measured the final thickness (±0.1 mm). Then, the specimens were cleaned ultrasonically (Quantrex 90, L&R Ultrasonic, Kearny, NJ, USA) in deionized water for 10 min and dried for 20 s before further evaluation. The disc specimens were then positioned over a spectrometer (MARC Resin Calibrator, Blue Light Analytics Inc., Halifax, NS, Canada) and light-cured for 20 s by direct application of the curing unit (Elipar TM S10, 3M, SP, Saint Paul, MN, USA) perpendicularly and positioned centrally over the specimen’s surface using a mechanical arm. During the specimen curing, a spectrometer measured the irradiance transmitted to the bottom of the specimens in real time. As a control, a ring mold without composite resin at different thicknesses was used. The MARC system contains a NIST-reference miniature spectrometer (USB4000, Ocean Optics, Dunedin, FL, USA) with a 3648-element linear CCD array detector (TCD1304AP, Toshiba, Tokyo, Japan). A CC3 cosine corrector is a sensor with a 4 mm diameter, designed to collect light radiation at around 180°, excluding optical interface problems, which are related to light collection sampling geometry. The irradiance at a wavelength of 360–540 nm was considered for data recording. 

### 2.7. Surface Analysis

Scanning electron microscopy (SEM, LEO, Oberkochen, Germany) and energy-dispersive spectroscopy (EDS, LEO, Oberkochen, Germany) provided the characterization of the microstructure and the major elemental composition of the material’s surface of the investigated 3D resins (magnification: 10,000×). Polished specimens (*n* = 2) from each composite were stored in a desiccator for one day. Then, they were coated with a gold layer using a sputter coater in a vacuum evaporator (BAL-TEC SCD 050 Sputter Coater, Liechtenstein) before SEM/EDS examination. SEM imaging was conducted using two electron accelerated voltages (20 and 2.70 Kv), working distances (13 and 4.9 mm), and aperture sizes (60 and 10 µm). The used image pixel size was 11.7 nm.

### 2.8. Statistical Analysis

The FS data underwent statistical analysis using SPSS version 29 (SPSS, IBM Corp. Armonk, New York, NY, USA), employing a two-way analysis of variance (ANOVA) and one-way ANOVA to analyze wear data at a significance level of *p* < 0.05. Subsequently, a Tukey HSD post hoc test was conducted to ascertain the distinctions among the groups.

## 3. Results

**Figure 2:**
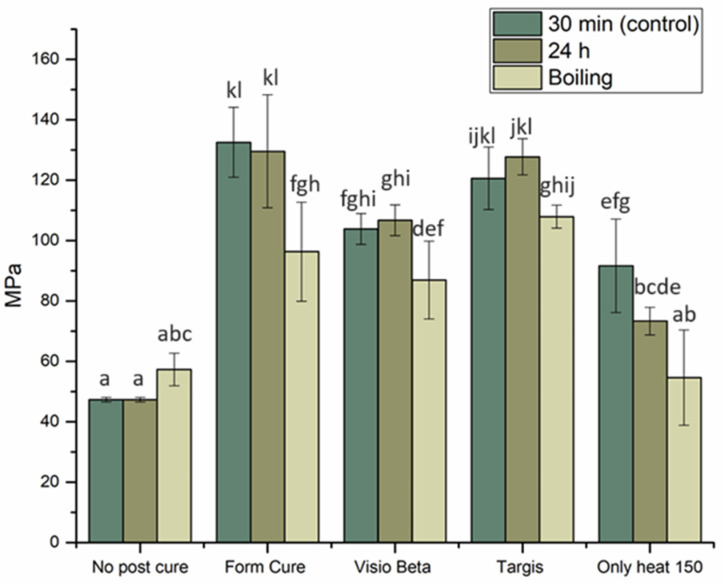
FS with SD of VSC specimens tested under different post-curing conditions. Different letters indicate statistical significance.

**Figure 3:**
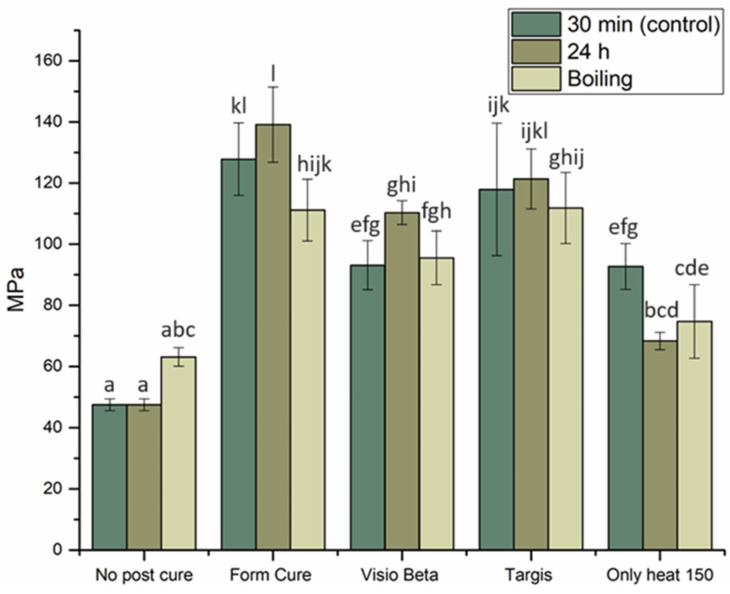
FS with SD of VST specimens tested under different post-curing conditions. Different letters indicate statistical significance.

The mean values of FS for VSC and VST materials with standard deviations (SDs) are summarized in Figure 2 and Figure 3. The VSC and VST specimens post-cured in the Form Cure unit and tested either immediately or after 24 h demonstrated the highest FS values. However, no statistically significant differences (*p* > 0.05) were observed when comparing these values to those of VSC specimens post-cured in the Targis curing unit and tested either immediately or after 24 h of dry storage or the VST specimens post-cured in the Targis curing unit and tested after 24 h. Conversely, both materials, when tested either immediately or after 24 h of dry storage without undergoing any post-curing treatment, demonstrated the lowest FS values. These differences were found to be statistically significant when compared with all other groups. Remarkably, following hydrothermal accelerated aging, the FS of these groups (no post-curing and VST-only heat group) were observed to surpass those without aging; however, the differences did not have statistical significance (*p* > 0.05). In all other groups, hydrothermal accelerated aging decreased the FS compared to the same materials subjected to the same post-curing methods.

Despite the observation that both materials subjected to post-curing by heat only exhibit higher FS compared to groups without post-curing, their FS remained among the lowest recorded within the tested groups.

**Figure 4:**
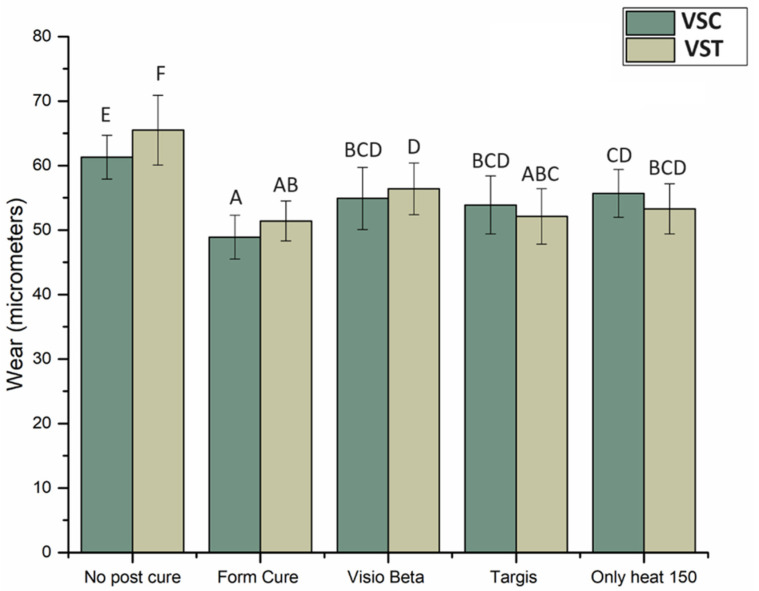
Wear depth in μm and SD of VSC and VST post-cured under different post-curing conditions. Different letters indicate statistical significance.

The mean wear depth values recorded for both materials after 15,000 chewing simulation cycles are presented in Figure 4. The data indicate that specimens of both VSC and VST post-cured in a Form Curing unit exhibited the lowest wear depth values compared to the other groups, with mean depths of 48.9 μm and 51.4 μm, respectively. In contrast, the VSC and VST groups that did not undergo post-curing exhibited the highest wear depths (*p* < 0.05) compared to other groups, with values of 61.3 μm and 65.5 μm, respectively. The remaining groups, as shown in Figure 4, exhibited nearly identical wear depth measurements.

**Figure 5:**
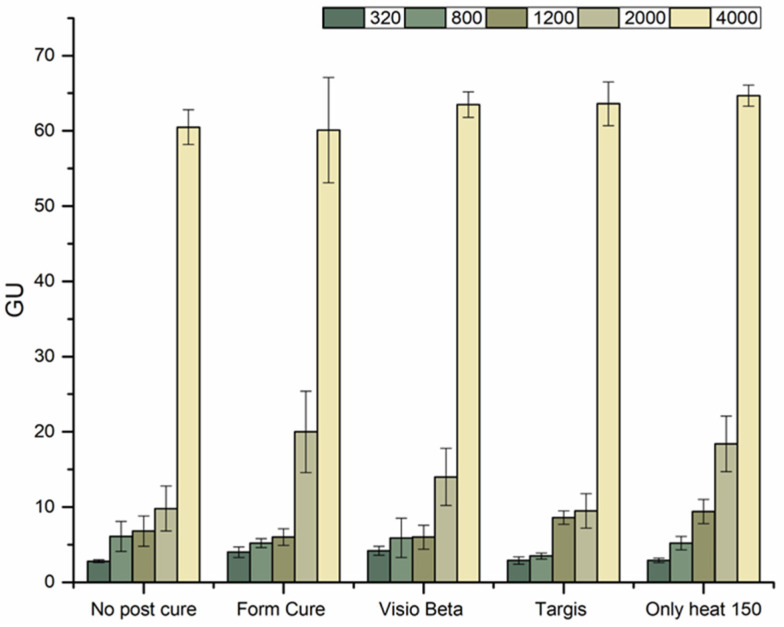
Surface gloss values of VSC specimens tested following polishing their surfaces using different silicon paper grit sizes (320, 800, 1200, 2000, and 4000).

Figure 5 shows the surface gloss measurements acquired from VSC specimens. Overall, employing silicon paper with a grit size of 4000 yielded the highest surface gloss irrespective of the post-curing method used, whereas silicon paper with a grit size of 320 yielded the lowest measurement.

**Figure 6:**
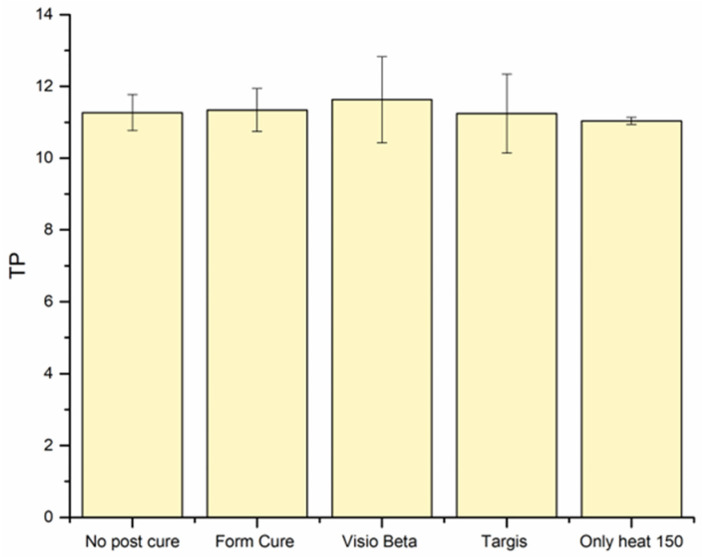
Translucency parameter (TP) mean values and standard deviations of VSC tested under different post-printing conditions.

Figure 6 shows the mean values and standard deviations of the translucency parameter (TP) for VSC specimens. Despite using various post-curing conditions, the TP values exhibited minimal variation, remaining largely consistent across the different conditions.

**Figure 7:**
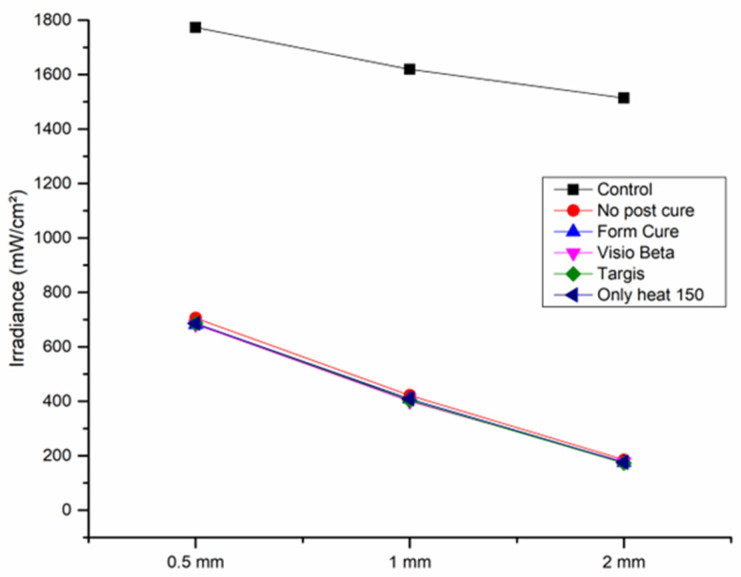
Light irradiance (mW/cm^2^) of the curing unit light at different thicknesses to the sensor through VSC specimens. Light curing without VSC specimens at different thicknesses was used as a control.

The irradiance values of transmitted light exhibit an inverse relationship with the thickness of the materials, with increasing thickness resulting in decreased light transmission. As illustrated in Figure 7, the quantity of light transmitted through specimens with a thickness of 2 mm was approximately half that of specimens with a thickness of 1 mm across all groups, and the transmission was highest through the 0.5 mm specimens. It is noteworthy that all experimental groups, irrespective of the post-curing condition employed, demonstrated equivalent light transmission values for each respective thickness. 

**Figure 8:**
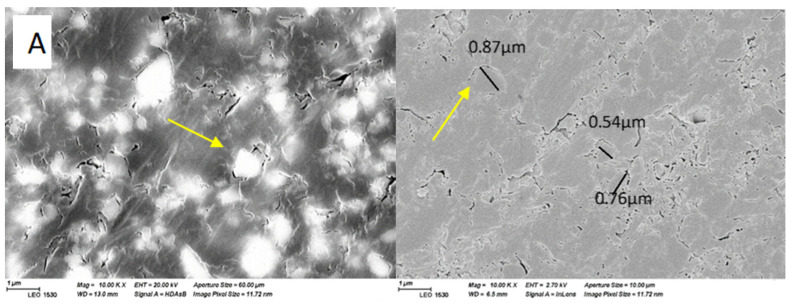

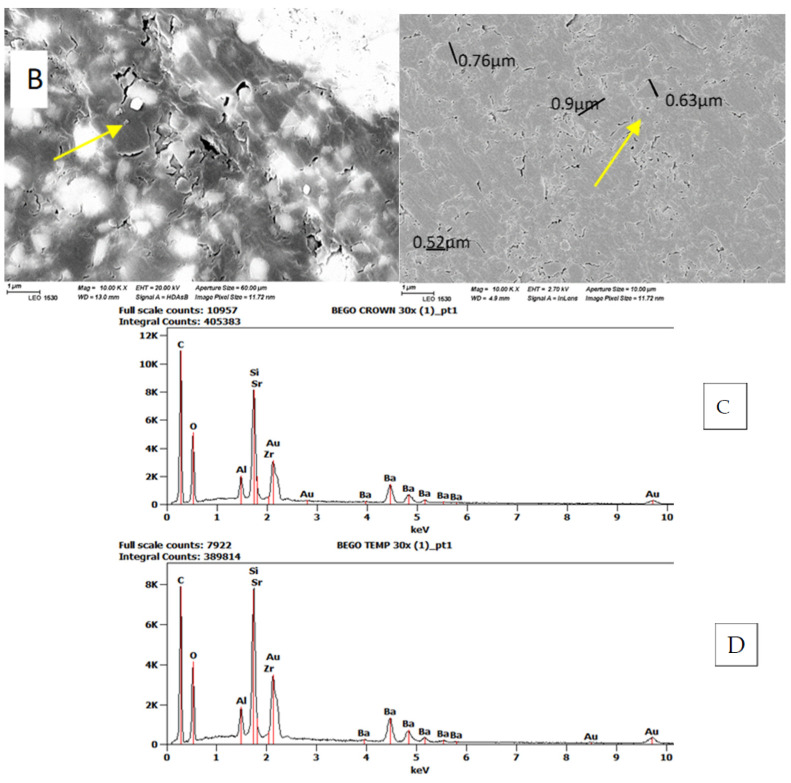
SEM images (10,000× magnification) of polished surfaces and EDS analyses of the investigated materials. (**A,C**) VSC. (**B,D**) VST.

The SEM analysis presented the microstructure of each tested material, with a similar appearance of the surface features (Figure 8). The ceramic particles can be seen (yellow arrows) as small fillers distributed within the resin matrix. The sizes of the ceramic fillers in both materials are observed to be predominantly smaller than one micrometer, as evidenced by the scale bars. This aligns with the manufacturer’s data indicating a filler size of 0.7 µm. EDS analysis indicated comparable levels of Si, Zr, Al, Ba, Sr, and Au in both VSC and VST materials. However, the C and O content was found to be more pronounced in VSC compared to VST (Table 2).

## 4. Discussion

According to the results, the post-printing conditions significantly affected the FS and surface wear but not the optical properties of materials under examination; hence, the null hypothesis was partially rejected.

The FS, denoting a material’s capacity to withstand bending or permanent deformation when subjected to an external load, stands as one of the crucial properties that reflects the mechanical characteristics of the examined material [15]. FS positively correlates with the degree of conversion (DC), which measures the extent of monomer conversion to polymer; as DC increases, so does the FS of the material [16]. An optimal wavelength during the curing process can effectively interact with the photoinitiators present in the resin, thereby enhancing the DC [17]. The printing unit used in this study (DLP) has a wavelength of 385 nm, which starts the polymerization reaction. However, during the printing process, complete polymerization of the photosensitive resin does not occur within the printer. Therefore, post-curing is recommended to enhance the DC and eliminate any remaining photoinitiator residue [18]. This was obvious from the results, as the groups of both materials that were not subject to post-curing treatment showed the lowest FS values. Moreover, the FS of groups subjected to post-curing exhibited significant variations based on the post-curing systems used. Particularly, the FS was significantly lower when cured by the Visio Beta than by the Form Cure and Targis units. This discrepancy may be attributed to the different wavelengths and temperatures from post-curing devices. According to the manufacturers, the Form Cure and Targis curing units exhibit respective wavelengths of 405 and 400–550 nm, with corresponding chamber temperatures of 60 and 40 °C, respectively. This combination along with a long post-curing time of 30 min might have positively influenced the degree of conversion, increasing the FS. Grzebieluch et al., in their investigation, examined the FS of VSC specimens subjected to post-curing using the Form Cure system. They reported a resulting FS value of 143.39 MPa, notably exceeding our obtained result of 132.5 MPa. This difference may arise from variations in the curing protocol employed, as a longer duration of curing was applied, with two cycles of 45 min each within the Form Cure system, potentially enhancing the degree of monomer conversion [19]. 

Interestingly, the groups that were subjected to only heat as a post-curing method resulted in comparatively lower FS values compared to those subjected to light-curing procedures. This can be explained by investigating the chemical composition of the tested materials, as both VSC and VST contain diphenyl (2,4,6-trimethyl benzoyl) phosphine oxide as a photoinitiator. Following a Norrish-type I mechanism, the photoinitiator within the photopolymer undergoes rapid homolytic cleavage upon ultraviolet (UV) irradiation, a process that is not feasible under heat-curing conditions [20]. Consequently, the resultant free radicals serve as initiators for the polymerization reaction within the resin matrix and increase the degree of conversion in favor of the light-curing methods [21,22]. 

The decrease in FS of 3D-printed materials following hydrothermal accelerated aging can be attributed to several underlying factors. Water absorption by the resin matrix can lead to hydrolytic degradation of the polymer network, causing a reduction in its mechanical properties. This degradation process can weaken the intermolecular bonds within the material, thereby compromising its structural integrity and consequently its FS [23]. Furthermore, water aging can also promote the leaching of unreacted monomers, residual solvents, and other by-products from the material, which can further compromise its mechanical properties [24]. These findings align with prior research, wherein it has been observed that the FS of 3D-printed materials is negatively influenced by artificial aging methods [25,26,27,28].

Two-body wear testing was used in the present study to investigate the impact of direct interaction between the specimen and the opposing element, which could occur during swallowing and parafunctional habits in addition to mastication. Specimens were subjected to 15,000 loading cycles during the wear test, a duration similar to short clinical usage; however, it was evidenced that approximately 40% of the total wear manifested within the initial 10,000 cycles in resin-based materials [29]. Following analysis of wear test outcomes, no significant differences in the wear values of the two materials were observed under the same post-curing conditions. According to the manufacturer, the two materials have similar compositions, including resin matrix constituents, filler types, and sizes. Therefore, it is unsurprising that the materials demonstrate nearly similar wear resistance under uniform post-curing conditions and test parameters. While there were minimal differences observed among the groups subjected to varying post-curing conditions, using Form Cure as a post curing method demonstrated superior wear resistance in both materials. This can also be attributed to the suitable wavelength and chamber temperature offered by the Form Cure unit, which facilitates the polymerization reaction. Heintze et al., in their review [30], explained a correlation between FS and wear, a finding confirmed by the present investigation.

As mentioned in the results, the VSC and VST groups that did not undergo post-curing showed the lowest resistance to wear compared to the other groups. These findings can be attributed to the lower hardness of these groups relative to other post-cured groups investigated in the study. Whether light- or heat-based, post-curing treatments facilitate further polymerization and crosslinking of the resin matrix, enhancing its mechanical and surface properties. Conversely, materials not undergoing post-curing exhibit incomplete polymerization, which make the material more susceptible to wear and degradation under mechanical stress [31]. A previous study [32] tested the wear characteristics of alternative 3D-printed materials, TEMP PRINT and GR-17 temporary, under identical wear testing conditions. The findings of the study revealed wear values ranging between approximately 20 and 30 μm, markedly lower than the values obtained in our study. This can be explained by the findings of Kessler et al. [33], where they highlighted the significance of filler size, shape, and volume to wear resistance, with the presence of smaller filler particles demonstrating enhanced wear resistance. According to the manufacturer, TEMP PRINT has nanometer-scaled fillers that can be removed together with the matrix, contributing to reduced wear values, while both VSC and VST fillers have bigger filler sizes, which could contribute to the higher wear values.

Gloss refers to a characteristic of visual presentation arising from how light is dispersed or reflected off a surface [34]. Following ISO standard 2813 [35] guidelines, determination of the gloss values of semigloss surfaces should include evaluation using an incident beam at a 60° angle, as applied in the present study. While prior research has demonstrated the influence of factors such as the DC on the gloss of resin-based materials [36], our results revealed that the primary determinant of the gloss values was the grit size of the silicon polishing paper. Remarkably, even groups not subjected to post-curing and that are conventionally associated with lower degrees of conversion exhibited gloss values comparable to other groups when polished with silicon papers of equivalent grit sizes. Consistent with the existing literature, dental professionals consider gloss levels ranging from 40 to 50 GU as clinically acceptable [32,37]. In alignment with these values, our findings indicate that polishing the surface using silicon paper with a grit size of 4000 was the only approach yielding gloss values within the acceptable range. Conversely, all other grit sizes resulted in gloss values below 20 GU, falling short of recommended clinical standards. 

The TP was proposed as a quantitative assessment of translucency and is characterized by the chromatic difference of a material at a specific thickness when positioned against standardized black and white backings [38]. In this study, TP was measured using the CIEL*a*b* color space model. The translucency values were assessed in 2 mm thick specimens, revealing minimal differences among the experimental groups, with TP mean values ranging from 11 to 11.6. Moreover, light transmission was mainly affected by the thickness of the specimen, which had an inverse relationship with the amount of the transmitted light. Interestingly, all groups, including the group without post-curing, exhibited comparable translucency and light transmission values. This suggests that factors beyond the degree of conversion may be at play in determining the optical properties. One of these factors is the filler content. The incorporated fillers significantly affect the light scattering, transmission, and translucency properties of resin materials [39]. Given that the barium glass fillers remained unaffected by post-printing conditions, it can be inferred that these fillers predominantly influenced the observed similar outcomes within different groups.

In a study by Pop-Ciutrila et al. [40], the average TP value for 2 mm thick specimens of human dentin was reported as 13.47. While direct comparisons across studies are constrained due to variations in experimental parameters, the TP values obtained for the 3D-printed materials in this study are approximate to those of human dentin. Translucency is linked to light scattering phenomena, with light transmission influenced by the refractive index mismatch between the filler and matrix constituents of resin-based materials [41]. This may explain the substantial differences between the TP and light transmission results of our study and the results observed in a separate study evaluating different 3D-printed materials under identical testing conditions [42]. VSC specimens exhibited elevated translucency parameters and transmission values compared to their TEMP PRINT and GR-17 counterparts, a phenomenon could be potentially attributed to the favorable alignment in refractive indices between the matrix and filler particles within the VSC material. 

SEM images (Figure 8) showed a similar microstructure of the tested materials; moreover, EDS analysis revealed comparable concentrations of Si, Zr, Al, Ba, Sr, and Au in both VSC and VST materials. Nevertheless, elevated levels of C and O were detected in VSC in contrast to VST. This difference may arise not only from variances in material composition but also from factors including sample heterogeneity and surface contaminations. Based on the available data regarding the chemical composition, both VSC and VST contain between 50–<75% by weight of monomers [9]. However, the exact monomer fraction for each material has not been precisely specified. Consequently, it could be that the actual monomer content in the two materials differs, which could account for the observed variations in carbon and oxygen content between them. The previous finding regarding similarity between the two investigated materials proposed an explanation for their nearly equivalent performance.

Given the manufacturer’s data and the results obtained from FS, wear, and SEM/EDS analysis, which suggest nearly identical behavior and compositions of the two tested materials, it prompts us to question the rationale behind classifying these materials into temporary and permanent categories. While this study provides insights into some of the tested materials’ properties, it is important to acknowledge several limitations. Though BEGO Otoflash (300–700 nm) or iLite Power, Fa. Heraeus Kulzer (390–540 nm) are the recommended light-curing systems for the tested materials, they were not used in this study and should be considered in future research. Additionally, given the expanding array of material options suitable for permanent applications, it is essential to note that this study did not include a comparison with other commercial materials for permanent use, which could provide further context and insights into the performance of the tested materials.

## 5. Conclusions

Within the limitations of this study, it could be concluded that the two tested materials exhibited nearly similar FS and wear behavior under similar post-printing conditions. Different post-printing treatment conditions significantly impacted the FS and surface wear resistance of these resins. The Form Cure device was the best post-curing unit compared to the other methods used; the specimens post-cured in this device showed the highest FS and lowest wear depth. Hydrothermal accelerated aging was found to decrease FS significantly in most cases. Optical properties remained nearly consistent across different post-printing conditions.

## Figures and Tables

**Table 1:** Materials used in the study.

Material	Manufacturer	Composition
VarseoSmile Crown plus (VSC)	BEGO Bremer Goldschlägerei Wilh. Herbst GmbH & Co. KG Bremen, Germany data	4.4′-isopropylidiphenol, ethoxylated, and 2-methylprop-2enoic acid. Silanized dental glass, methyl benzoylformate, diphenyl (2,4,6-trimethylbenzoyl) phosphine oxide. Inorganic filler particle size of 0.7 μm, 30–50% by wt.
VarseoSmile Temp (VST)		4.4′-isopropylidiphenol, ethoxylated, and 2-methylprop-2enoic acid. Silanized dental glass, methyl benzoylformate, diphenyl (2,4,6-trimethylbenzoyl) phosphine oxide. Inorganic filler particle size of 0.7 μm, 30–50% by wt.

**Table 2:** Atomic percentage of major elemental compositions of investigated materials determined with EDS analysis.

Materials	Atomic Percentage
VSC	C 56.47%, O 31.99%, Al 1.59%, Si 8.09%, Sr 0.09%, Ba 1.77%
VST	C 55.43%, O 30.56%, Al 1.88%, Si 9.80%, Sr 0.15%, Ba 2.19%

## Data Availability

The original contributions presented in the study are included in the article, further inquiries can be directed to the corresponding author.
